# Cytokines as Biomarkers of Treatment Response to IFN***β*** in Relapsing-Remitting Multiple Sclerosis

**DOI:** 10.1155/2014/436764

**Published:** 2014-07-22

**Authors:** Nikolaos Dimisianos, Maria Rodi, Dimitra Kalavrizioti, Vasileios Georgiou, Panagiotis Papathanasopoulos, Athanasia Mouzaki

**Affiliations:** ^1^Department of Neurology, Patras University Hospital, 26500 Patras, Greece; ^2^Division of Hematology, Department of Internal Medicine, Medical School, University of Patras, 26500 Patras, Greece; ^3^Hellenic Open University, Parodos Aristotelous 18, 26335 Patras, Greece

## Abstract

*Background*. MS patients show a remarkable heterogeneity in their response to disease modifying treatments. Given the need for early treatment initiation and the diversity of available options, a predictive marker that indicates good or poor response to treatment is highly desirable. *Objective*. To find a biomarker for treatment response to IFN*β* among pro- and anti-inflammatory cytokines. *Materials and Methods*. IFN-*γ*, TNF-*α*, IL-2, IL-4, IL-6, IL-10, IL-17A, and TGF-*β*1 levels were measured in serum and CSF of 43 patients with RR-MS who were followed up for a mean period of 5.3 years. Thirty-five patients received IFN*β* treatment and were divided into good responders (GR, *n* = 19) and poor responders (PR, *n* = 16). The remaining 8 patients showed a very favorable outcome and remained untreated (noRx). *Results*. GR had significantly higher serum baseline levels of IL-17A than PR and significantly higher serum levels of IL-17A, IFN-*γ*, TNF-*α*, and IL-2 than noRx. PR had significantly higher IFN-*γ* serum levels than noRx. No significant differences were observed in serum levels of IL-6, IL-4, IL-10, and TGF-*β*1 or the levels of all cytokines measured in CSF between the 3 groups of patients. *Conclusions*. Baseline serum levels of IL-17A can be used as a biomarker of IFN*β* treatment response.

## 1. Introduction

Multiple sclerosis (MS) is a chronic, demyelinating disease of the central nervous system (CNS), affecting young adults with a female preponderance [1]. At an individual level, MS is extremely heterogeneous in its initial presentation, rate and severity of relapses, pattern of disease progression, underlying immunopathology, radiological appearance, and response to the disease modifying treatments (DMTs) [2]. In the last 20 years the initial paucity of available, approved drugs for MS prophylaxis has been replaced by a considerable number of treatment options. The first DMTs approved for relapsing-remitting MS (RR-MS) prophylaxis were the interferons-beta (IFN*β*) and glatiramer acetate (GA), which remain first-line drugs, followed by other agents like natalizumab, mitoxantrone, and fingolimod, assigned as second-line treatments. The availability of multiple DMTs and the need for early initiation of prophylactic treatment have rendered prognostic markers of treatment-response highly desirable.

Patients with MS receiving DMTs can be classified as good responders (slower disease progression or lower relapse rate), partial responders, and nonresponders (no effect on disease activity) [3]. Although there are many ways to assess disease activity and treatment response, including clinical, radiological, neurophysiological, and molecular, the concept of treatment response is mainly based on the clinical expression of MS, that is, relapses and disease progression. Clinical criteria for treatment response are still uncertain and are usually extrapolated from natural history studies and placebo cohorts of clinical trials of drugs [4].

In clinical trials, IFN*β* has demonstrated efficacy in RR-MS patients by reducing relapse rate, lesion formation in the CNS, and progression of sustained disability [5–7]. However, IFN*β* therapy is not efficacious in a significant proportion of patients. In two studies comparing three IFN*β* preparations (IFN*β*-1*α* 30 *μ*g qw versus IFN*β*-1*β* 250 *μ*g qod and IFN*β*-1*α* 44 *μ*g tiw versus IFN*β*-1*α* 30 *μ*g qw), the percentage of patients that remained relapse-free in the 2-year follow-up ranged between 36 and 51% [8, 9]. The percentage of active T2 lesion-free patients ranged between 26 and 58% and of EDSS progression-free patients between 70 and 87% [8, 9].

The factors that determine individual response to IFN*β* remain elusive. Proposed markers of poor prognosis include ongoing MRI activity for 1-2 years after treatment initiation, with relapses and/or disease progression, or the development of neutralizing antibodies against IFN*β* [10–12]. Nevertheless, these markers require that IFN*β* is initiated and taken for a considerable time period before they can be assessed. An ideal marker should predict treatment response before treatment initiation.

The advantages of using cytokines as biological markers of MS are that they reflect the underlying immunopathology in the periphery (blood) and/or the CNS (CSF), are easily measured, and are most likely to be affected by DMTs that target the immune component of MS pathogenesis. Several studies have investigated whether interleukin-17 (IL-17) can be used as a biological marker of response to IFN*β*, with conflicting results [13–16]. The focus on IL-17 as a potential biomarker followed the discovery of IL-17 producing T-cells (Th17) as key players in the pathogenesis of MS [17, 18]. However, the immune pathogenesis of MS is far from straightforward with many components of the innate and adaptive immune system participating and interacting in a complex manner [19, 20].

The aim of our study was to expand the search for prognostic biomarkers of treatment response to IFN*β* among pro- and anti-inflammatory cytokines in the serum and CSF of RR-MS patients who were prospectively followed up for a considerable time period (mean 5.3 years).

## 2. Materials and Methods

### 2.1. Patients

The patients included in this study were recruited from a single MS center (Neurology Clinic of Patras University Hospital). They were hospitalized with symptoms suggestive of MS and underwent a thorough diagnostic workup, which included brain and spinal cord MRI, lumbar puncture for CSF analysis (including detection of oligoclonal bands (OCB) and/or IgG index calculation), visual evoked potentials (VEP), and other laboratory tests necessary for the exclusion of other diagnoses (e.g., systemic lupus erythematosus, vasculitis, infections, vitamin B12 deficiency, etc.). A detailed history was taken, for information about symptoms in the past that could be attributed to MS, estimation of disease duration, concomitant illnesses, and family history, and a detailed neurological examination was performed to determine the patients' level of disability according to the Expanded Disability Status Scale (EDSS) [21]. Exclusion criteria included diagnoses other than MS, active infection or inflammation of any kind, and current or recent treatment with immunosuppressive or immunomodulatory drugs. A total of 43 patients, who were eventually diagnosed as having RR-MS, according to the 2005 McDonald diagnostic criteria [22], were included in the study. The study protocol was noninterventional, meaning that all patient-related decisions such as the time and type of treatment, follow-up schedule, and repeat MRIs were made by the patients' treating physicians. All patients received a 3-day corticosteroid treatment (1 g/day of methylprednisolone, iv) during their hospitalization. Blood and CSF samples were collected prior to corticosteroid treatment and before initiation of IFN*β* treatment.

Patients were prospectively followed up with visits twice a year (approximately every 6 months), except for the first visit after enrollment, which was performed as soon as the patients entered remission (usually 1-2 months after baseline). In each follow-up visit, information about relapses since the last visit, current medication, and results of repeat MRIs were collected, and a neurological examination was performed for determination of the EDSS score. At the end of the follow-up period, patients were characterized as poor responders (PR) to DMTs if they had a sustained increase in EDSS score of ≥1 point in a 2-year period and/or an annualized relapse rate (ARR) of ≥1. Patients who did not fulfill these criteria were characterized as good responders (GR). The treatment response criteria were applied during the period in which the patients were under IFN*β* treatment (Table 3). The study started on January 2005 (first patient, first visit) and ended on February 2013 (last patient, last visit).

All subjects gave written informed consent before enrollment in the study. The study protocol was approved by the Patras University Hospital Ethics (Re: 296) and Scientific (Re: 451) Committees as part of a general application to collect biological samples from patients attending the Neurology Clinic to study in vitro the role of T helper cell populations and cytokines in the pathogenesis, prognosis, and natural course of multiple sclerosis. The Hospital abides by the Helsinki declaration on ethical principles for medical research involving human subjects.

### 2.2. Determination of Cytokine Levels

Serum and CSF samples from patients and controls were collected and stored at −75°C until processing. Determination of serum and CSF levels of the cytokines IFN-*γ*, TNF-*α*, IL-2, IL-4, IL-6, IL-10, and IL-17A was performed on a BD FACSArray Bioanalyzer using the Cytometric Bead Array (CBA) assay (human Th1/Th2/Th17 Cytokine Kit, BD Biosciences, San Diego, USA). Serum and CSF levels of TGF-*β*1 were measured by ELISA (R&D Systems Quantikine TM, Minneapolis, MN, USA). The data were analyzed using the CurveExpert V1.4. Cytokine ratios (Th1/Th2, Th1/Th17, Th17/Th2, Type-1/Type-2, IFN-*γ*/IL-10, and IL-17A/IL-10) were also calculated (Table 1).

### 2.3. Statistical Analysis

Cytokine levels in serum and CSF, the various cytokine ratios, and patient data were compared between the patient groups using the Kruskal-Wallis nonparametric statistical hypothesis test because the application of Shapiro-Wilk normality test showed that in most cases of the dataset the normality assumption does not hold. When the null hypothesis of the Kruskal-Wallis test was rejected, the Mann-Whitney test with Bonferroni corrections was employed for the pairwise comparisons of the groups. Differences between groups were considered significant if *P* was ≤0.05. Data were analyzed using the GraphPad Prism v. 5.03 (San Diego, CA, USA).

## 3. Results

### 3.1. Patient Follow-Up and Allocation to Treatment Response Groups

Table 2 shows the data of the study subjects, including their clinical and laboratory parameters. The patients were followed up for a mean period of 5.3 years. Eight out of the 43 patients who completed the follow-up period did not receive any DMTs (noRx). The remaining 35 patients received treatment with at least one of the IFN*β* formulations (IFN*β*-1*α* im, Avonex, *n* = 11, IFN*β*-1*α* sc, Rebif, *n* = 12, IFN*β*-1*β* sc, Betaferon, *n* = 12) at standard doses. Those who, according to the assessment of their treating physician, did not respond well to IFN*β* switched to GA or a second-line therapy (fingolimod or natalizumab). Accordingly, 3 patient groups were formed: the 1st included patients with good response to IFN*β* (GR, *n* = 19), the 2nd included patients with poor response to IFN*β* (PR, *n* = 16), and the 3rd included patients who received no treatment (noRx, *n* = 8) (Table 3).

The noRx patients had a significantly milder disease compared to the other groups (lower EDSS scores from baseline to the end of the follow-up period, no or rare relapses) and significantly fewer Gd-enhancing lesions on baseline MRI and cells in the CSF. The main reasons these patients did not receive a prophylactic treatment included milder disease at presentation with a more benign course and the patients' own preference. GR patients, compared to PR, had lower EDSS score, which became statistically significant early on, since the initial remission (*P* = 0.01). The main criteria of treatment response, that is final EDSS score and ARR under IFN*β* treatment, were, as expected, much higher in the PR patients compared to GR (*P* = 0.001 and *P* < 0.0001, resp.). All other parameters, like age at diagnosis, disease duration, pre-IFN*β* ARR, IgG index and cells in the CSF, lesions at baseline MRI, and total follow-up duration, did not differ significantly between the two groups. In the PR group, the numbers of female and male patients were equal, whereas in the GR and noRx groups there was a higher female to male ratio, although the difference did not reach statistical significance. The duration of IFN*β* treatment was significantly shorter in the PR group (*P* = 0.05), a finding that was expected since PR patients were more likely to switch earlier to an alternative DMT (Table 3).

The proportion of relapse-free patients at the end of the follow-up period was 87.5% for noRx, 58.8% for GR, and 0% for PR patients. Similarly, the proportion of patients free of EDSS progression (>1 point) was 100% for noRx, 94.1% for GR, and 33.3% for PR patients (Table 3).

### 3.2. Serum and CSF Cytokines by Treatment Response Group

**Figure 1:**
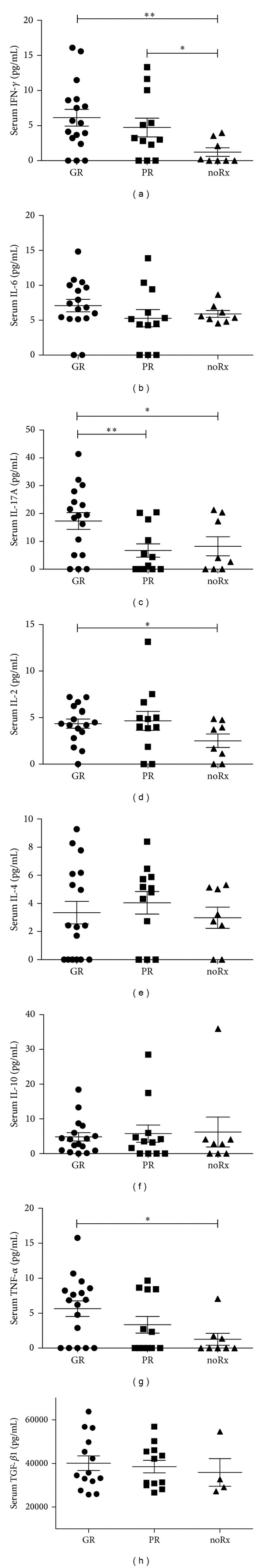
Serum cytokines in GR, PR, and noRx patients. (a) IFN-*γ*, (b) IL-6, (c) IL-17A, (d) IL-2, (e) IL-4, (f) IL-10, (g) TNF-*α*, and (h) TGF-*β*1. *P = 0.05, **P = 0.03.

GR patients had significantly higher serum levels of IL-17A compared to PR and noRx patients (*P* = 0.03 and *P* = 0.05, resp.) and significantly higher serum levels of IFN-*γ* (*P* = 0.03), TNF-*α* (*P* = 0.05), and IL-2 (*P* = 0.05) compared to noRx patients (Figure 1). PR patients had significantly higher IFN-*γ* serum levels than noRx patients (*P* = 0.05) (Figure 1). No significant differences were observed in serum levels of IL-6, IL-4, IL-10, or TGF-*β*1 between the 3 groups of patients (Figure 1). CSF cytokine levels did not show statistically significant differences between the 3 groups of patients (see Tables  S1 and S2 in Supplementary Materials available online at http://dx.doi.org/10.1155/2014/436764).

### 3.3. Serum and CSF Cytokine Ratios by Treatment Response Group

**Figure 2:**
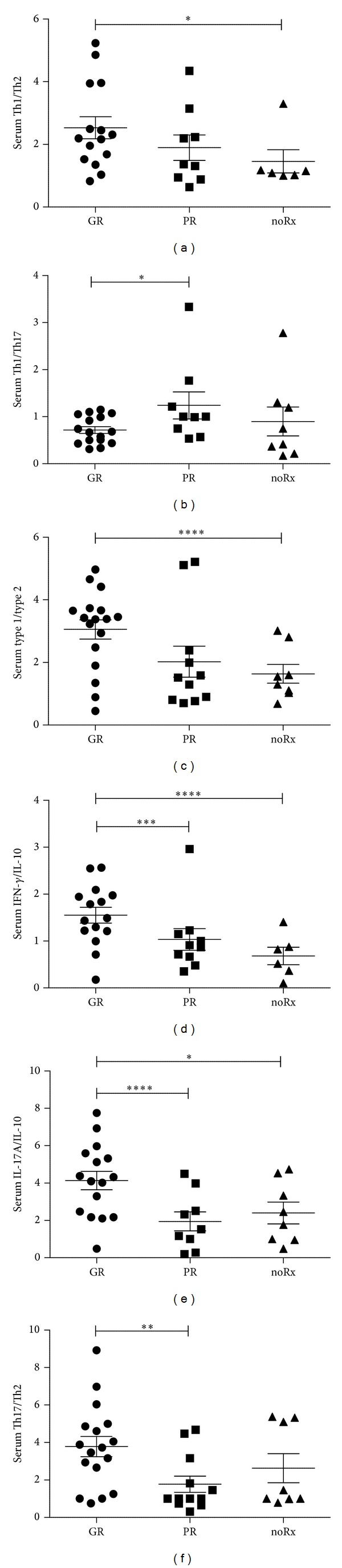
Serum cytokine ratios in GR, PR, and noRx patients. (a) Th1/Th2, (b) Th1/Th17, (c) Type 1/Type 2, (d) IFN-*γ*/IL-10, (e) IL-17A/IL-10, and (f) Th17/Th2. *P = 0.05, **P = 0.03, ***P = 0.02, ****P = 0.01.

GR patients had significantly higher IL-17A/IL-10 (*P* = 0.01) and Th17/Th2 ratios (*P* = 0.03) and a lower Th1/Th17 ratio (*P* = 0.05) of serum cytokines than PR patients, reflecting their higher serum levels of IL-17A (Figure 2). GR patients had also a significantly higher IFN-*γ*/IL-10 ratio of serum cytokines than PR patients (*P* = 0.02), whereas the Th1/Th2 and Type 1/Type 2 serum cytokine ratios were not statistically different between the two groups (Figure 2). GR patients had significantly higher Th1/Th2 (*P* = 0.05), Type 1/Type 2 (*P* = 0.01), IL-17A/IL-10 (*P* = 0.05), and IFN-*γ*/IL-10 (*P* = 0.01) serum cytokine ratios than noRx patients (Figure 2). The differences in serum cytokine ratios between PR and noRx patients were not statistically significant (Figure 2). Differences in cytokine ratios in the CSF between the 3 groups were not statistically significant (Tables  S3 and S4).

The calculations of Type 1/Type 2 cytokine ratios were performed with or without the inclusion of IL-2 values in the nominator, with no significant alterations in the outcome (cf. Table 1 and Tables S3 and S4).

## 4. Discussion

Results from clinical trials have shown that IFN*β* preparations slow down disease progression and reduce relapse rates in RR-MS patients by an average of 30% [5–7, 23]. The causes for poor response to IFN*β* in a percentage of patients remain obscure. It has been suggested that they may be attributed to the development of neutralizing antibodies [12], or, as it can be inferred from a recent study [24], to failure of induction of a new regulatory T-cell population, the FoxA1+ regulatory T-cells, that are induced by IFN*β* and are found in MS patients that responded to IFN*β* therapy.

In our study, we searched for prognostic biomarkers of treatment response to IFN*β* among pro- and anti-inflammatory cytokines in the serum and CSF of RR-MS patients who were prospectively followed up for a considerable time period (mean 5.3 years). We also calculated various cytokine ratios, to assess the relative concentrations of antagonizing cytokines, as expressed with calculated ratios that reflect the profiles of T helper cells (Th1/Th2, Th1/Th17, Th17/Th2, IFN-*γ*/IL-10, and IL-17A/IL-10), or an overall picture of the immune response (Type-1/Type-2) since it includes cytokines the expression of which is not restricted to specific cell populations [25, 26]. Type 1/Type 2 ratios were calculated with and without the values for IL-2 concentrations because IL-2 is a pleiotropic cytokine that, in humans, is secreted by naive Th cells when activated, stimulates proliferation and effector functions of Th, cytotoxic T-cells, B-cells, and NK cells, promotes activation-induced cell death, but it also suppresses Th17 differentiation and is an essential growth factor of regulatory T-cells [27, 28].

Serum IL-17A was the main cytokine that distinguished GR from PR or noRx patients, and this difference was confirmed by the cytokine ratios Th1/Th17, IL-17A/IL-10, and Th17/Th2. Serum IFN-*γ* was the only cytokine that distinguished PR patients from noRx patients who, otherwise, presented with the lowest levels of proinflammatory cytokines. We found no significant differences in the levels of anti-inflammatory cytokines in serum or in the levels of all cytokines measured in the CSF of GR, PR, or noRX patients.

Several studies have shown that the beneficial effects of IFN*β* are, at least partially, mediated through reduction of IL-17 [13, 29–31], which could explain the favorable response of patients with higher baseline IL-17A observed in our study.

Durelli et al. [13] showed that RR-MS patients with active disease have a significantly higher percentage of Th17 cells than patients with inactive disease and that IFN*β* decreases Th17 cells, but not Th1 cells. The effect of IFN*β* was attributed to increased expression of IFN-*α*R1 on Th17 cells causing a stronger IFN*β*-dependent STAT1 phosphorylation, leading to apoptosis [13]. Ramgolam et al. [31] showed that IFN*β* downregulates IL-1*β* and IL-23p19 gene expression whereas it upregulates IL-12p35 and IL-27p28 in MS patients' dendritic cells, leading to suppression of Th17 differentiation. They also found a direct effect of IFN*β* on T-cells, mediated by inhibition of RORc, IL-17A, and IL-23R gene expression and by upregulation of IL-10 gene expression [31].

On the other hand, Axtell et al. [14] found that a subset of nonresponders to IFN*β* had high pretreatment serum levels of IL-17F and endogenous IFN*β*, compared to responders. This, according to the authors, could be explained by the hypothesis that this subset of patients had aggressive Th17 disease, which the immune system tried to counteract by upregulating endogenous IFN*β*; thus, the addition of exogenous IFN*β* could not be effective [14]. The notion that high pretreatment levels of IL-17 are a prognostic biomarker of poor response to IFN*β* could not be confirmed by subsequent studies [16, 32, 33].

In our study blood and CSF samples were collected during MS exacerbation (ongoing symptoms, evidence of active lesions on MRI), as opposed to most relevant studies [14, 16, 32, 33]. Since cytokines are short-lived molecules and the immunological reactions are most evident during the inflammatory, acute phase of the disease, measuring cytokines at this phase depicts more accurately the ongoing immune response. A potential pitfall is that cytokine levels measured in blood samples collected from MS patients during exacerbations may be influenced by a concurrent systemic infection, a phenomenon quite common, as shown by Buljevac et al. [34]. In addition, the follow-up period of the patients is the longest in any other study that addressed treatment response, giving the opportunity for a better assessment of disability progression and relapse rates in the long-term.

Treatment response is a controversial issue and remains a matter of debate [35]. Placebo cohorts of clinical trials have been very diverse regarding ARR, ranging from 1.28 in the earlier IFN*β* pivotal trials [5–7] to as low as 0.39 in the more recent laquinimod or BG-12 clinical trials [36, 37].

Natural history studies, performed before the advent of DMTs, provide a clearer picture of the long-term course of the disease in terms of relapse rates and progression. Such studies indicate an average ARR of around 0.5, which is even higher when longitudinal, prospective assessments are considered [4]. The application of the treatment response criteria set in our study (an ARR of ≥1 and a sustained increase in EDSS of ≥1 over a period of 2 years indicating poor response) provided a clear-cut difference between GR and PR patients. GR patients had a mean ARR under IFN*β* of 0.18 and a progression of disability of only 0.23 points in the EDSS during the follow-up period (mean 5.07 years), whereas PR patients had a mean ARR of 1.42 and an increase in EDSS of 1.78 (mean 5.39 years) (Table 3). Since our study was not interventional, MRI scans were performed at a frequency defined by each treating physician. These MRI scan results were made available to us and were taken into consideration when the patients were characterized as having had a relapse or not since their previous visit.

Benign MS is another controversial issue, with many definitions and criteria, the most common being an EDSS of ≤3 after 10 years of disease duration [4, 38]. The subgroup of patients with no treatment (noRx) of our study fits this definition, because their total mean disease duration (from onset of symptoms until the end of the follow-up period) exceeded 10 years and the mean EDSS score at the end of the study was 1.12. They also had almost no relapses during the follow-up period (only one patient had a relapse, with no residual deficit, mean ARR 0.03) (Table 3). This group of patients had the lowest levels of nearly all serum proinflammatory cytokines and of the corresponding cytokine ratios (Tables  S1–S4), and the fewest gadolinium-enhancing lesions on MRI and inflammatory cells in the CSF.

In the new era of multiple available treatment options for RR-MS, a reliable, easily measured biomarker of treatment response is clearly needed. In our study we searched for biomarkers for the prediction of response to IFN*β* and of a benign disease course, among serum and CSF pro- and anti-inflammatory cytokines. Our results indicate that (i) baseline serum IL-17A levels distinguish GR from PR patients, (ii) serum IFN-*γ* levels distinguish PR from noRx patients, and (iii) noRx patients (with minimal disability and a benign course) also exhibit minimal inflammation. The cytokine profiles of GR patients versus PR/noRx patients discriminate the latter groups as those who should obviate treatment with IFN*β*. Our results need, of course, to be validated in a larger cohort of patients and for other DMTs.

## Supplementary Material

Supplementary Tables S1-S4: contain the complete sets of data from all the experiments (given as mean±SD), including the values of CSF cytokine levels and ratios that are not shown in the main body of the manuscript.

## Figures and Tables

**Table 1:** Cytokines and cytokine ratios measured in the serum and CSF of RRMS patients.

Cytokines	IFN-*γ*, TNF-*α*, TGF-*β*1, IL-2, IL-4, IL-6, IL-10, IL-17A
Th1/Th2	[IFN-*γ* + TNF-*α*]/IL-4
Th1/Th17	[IFN-*γ* + TNF-*α*]/IL-17A
Th17/Th2	IL17A/IL-4
Type 1/Type 2	[IFN-*γ* + TNF-*α* + IL-17A + IL-6 ± IL-2∗]/[IL-4 + IL-10 + log⁡TGF-*β*1]
Other cytokine ratios	IFN-*γ*/IL-10, IL-17A/IL-10

*Type 1/Type 2 ratios were calculated with and without the values for IL-2 concentrations.

**Table 2:** Data of study subjects.

Patients (*n*)	43
Female/male (*n*)	25/18
Age at diagnosis (years)	33.26 ± 9.88
Disease duration (years)	2.22 ± 3.35
EDSS acute	2.72 ± 0.95
EDSS remission	1.93 ± 0.96
EDSS final	2.66 ± 1.90
ARR (total)	0.43 ± 0.56
Follow-up duration (years)	5.31 ± 1.58
IgG index	1.02 ± 0.62
T2 lesions (baseline)	10.51 ± 5.52
T1 Gd+ lesions (baseline)	2.16 ± 2.12
CSF cell number (per *μ*L)	9.72 ± 8.29

Data are presented as mean ± SD; T1 Gd+: gadolinium enhancing lesions.

**Table 3:** Data of study subjects separated in treatment response groups.

	GR (*n* = 19)	PR (*n* = 16)	*P*	noRx (*n* = 8)	*P* (versus GR, PR)
Baseline characteristics					
Female/male (*n*)	12/7	8/8	NS∗	5/3	
Age at diagnosis (years)	31.42 ± 10.25	32.88 ± 10.82	0.68	38.38 ± 5.09	0.24, 0.36
Disease duration (years)	1.28 ± 2.36	2.31 ± 2.97	0.21	4.27 ± 5.18	0.18, 0.47
Pre-IFN*β* ARR	1.46 ± 1.08	1.61 ± 0.52	0.45	0.54 ± 0.26	**0.006, 0.003**
T2 lesions	9.68 ± 6.17	12.31 ± 5.12	0.18	8.87 ± 4.08	0.73, 0.11
T1 Gd+ lesions	2.73 ± 2.30	2.31 ± 2.05	0.65	0.50 ± 0.53	**0.01, 0.05**
IgG index	1.11 ± 0.54	1.02 ± 0.76	0.39	0.85 ± 0.52	0.13, 0.31
CSF cells (per *μ*L)	11.16 ± 8.65	10.81 ± 8.16	0.90	4.12 ± 5.79	**0.05, 0.01**
EDSS acute	2.74 ± 0.93	3.15 ± 0.89	0.27	1.81 ± 0.37	**0.01, 0.003**

Follow-up data					
Follow-up duration (years)	5.07 ± 1.57	5.39 ± 1.58	0.55	5.71 ± 1.53	0.32, 0.71
EDSS remission	1.79 ± 1.01	2.40 ± 0.93	**0.01**	1.31 ± 0.25	0.30, **0.003**
EDSS final	2.02 ± 1.39	4.18 ± 1.87	**0.001**	1.12 ± 0.23	**0.01, 0.001**
IFN*β* duration (years)	4.01 ± 1.55	2.91 ± 1.68	**0.05**	NA	
ARR under IFN*β*	0.18 ± 0.27	1.42 ± 0.75	**<0.0001**	(0.03 ± 0.09)^§^	
Relapse free (%)	58.82%	0%	**<0.0001**	87.5%	**0.003, <0.0001**
EDSS progression-free (%)	94.11%	33.33%	**0.01**	100%	0.60, **0.001**

Data are given as mean ± SD; ARR: annualized relapse rate; GR: good response; PR: poor response, noRx: no treatment; NA: not applicable; NS: not significant (∗chi-square test); ^§^corresponds to ARR for the entire follow-up period under noRx; numbers in bold denote statistical significance.
