# Supplementary material for: Cytokines as Biomarkers of Treatment Response to IFNβ in Relapsing-Remitting Multiple Sclerosis
Source: Mult Scler Int. 2014 Jul 22;2014:436764. doi: 10.1155/2014/436764 (PMC4134814; doi:10.1155/2014/436764)
Supplement: Supplementary file 1 — Supplementary Tables S1-S4: contain the complete sets of data from all the experiments (given as mean±SD), including the values of CSF cytokine levels and ratios that are not shown in the main body of the manuscript. [file 436764.f1.pdf]

**Supplementary Tables S1 and S2.** Serum and CSF cytokine levels of MS patients separated by treatment response group

| **Cytokine in serum** | **GR** | **PR** | **p** | **noRx** | **p (vs GR, PR)** |
|---|---|---|---|---|---|
| **IFN-γ** | 6.12 ± 4.90 | 4.73 ± 4.59 | 0.44 | 1.22 ± 1.72 | **0.03, 0.05** |
| **IL-6** | 7.09 ±3.71 | 5.28 ± 4.29 | 0.23 | 5.89 ± 1.35 | 0.38, 0.70 |
| **IL-17A** | 17.32 ± 12.33 | 6.67 ± 8.37 | **0.03** | 8.19 ± 9.64 | **0.05**, 0.81 |
| **IL-2** | 4.34 ± 2.04 | 4.65 ± 3.53 | 0.77 | 2.50 ± 2.04 | **0.05**, 0.13 |
| **IL-4** | 3.33 ± 3.29 | 4.04 ± 2.77 | 0.57 | 2.98 ± 2.15 | 0.95, 0.37 |
| **IL-10** | 4.81 ± 4.98 | 5.75 ± 8.63 | 0.54 | 6.20 ±12.45 | 0.30, 0.66 |
| **TNF-α** | 5.63 ± 4.59 | 3.48 ± 4.13 | 0.32 | 1.26 ± 2.44 | **0.05**, 0.29 |
| **TGF-β1** | 40,094 ± 12,414 | 38,496 ± 10,017 | 0.77 | 35,858 ± 12,660 | 0.42, 0.67 |

| **Cytokine in CSF** | **GR** | **PR** | **p** | **noRx** | **p (vs GR, PR)** |
|---|---|---|---|---|---|
| **IFN-γ** | 3.56 ± 5.52 | 4.80 ± 7.19 | 0.90 | 4.57 ± 5.48 | 0.32, 0.30 |
| **IL-6** | 5.56 ± 6.64 | 5.99 ± 4.22 | 0.46 | 6.47 ± 4.10 | 0.30, 0.80 |
| **IL-17A** | 8.33 ± 12.20 | 14.48 ± 18.42 | 0.48 | 17.02 ± 16.38 | 0.17, 0.63 |
| **IL-2** | 1.13 ± 1.91 | 2.49 ± 3.34 | 0.30 | 2.30 ± 1.72 | 0.06, 0.66 |
| **IL-4** | 2.24 ± 2.67 | 2.30 ± 2.01 | 0.63 | 2.81 ± 2.82 | 0.71, 0.64 |
| **IL-10** | 2.30 ± 4.17 | 4.49 ± 6.61 | 0.67 | 2.92 ± 1.66 | 0.06, 0.58 |
| **TNF-α** | 2.62 ± 3.53 | 2.39 ± 3.53 | 0.83 | 5.25 ± 4.95 | 0.19, 0.28 |
| **TGF-β1** | 79.37 ± 20.18 | 67.29 ± 48.87 | 0.25 | 67.43 ± 31.44 | 0.47, 0.79 |

Data are given as mean ± SD; cytokines were measured in pg/ml; numbers in bold denote statistical significance.

**Supplementary Tables S3 and S4.** Serum and CSF cytokine ratios in MS patients separated by treatment response group

| Serum cytokine ratios | GR | PR | p | noRx | p (vs GR, PR) |
|---|---|---|---|---|---|
| **Th1/Th2** | 2.53± 1.35 | 1.89 ± 1.22 | 0.26 | 1.45 ± 0.90 | **0.05**, 0.68 |
| **Th1/Th17** | 0.71 ± 0.29 | 1.24 ± 0.86 | **0.05** | 0.89 ± 0.87 | 0.88, 0.24 |
| **Type 1[a]/Type 2** | 3.05 ± 1.27 | 2.02 ± 1.64 | 0.07 | 1.63 ± 0.84 | **0.01**, 0.54 |
| **Type 1[b]/Type 2** | 2.69 ± 1.19 | 1.54 ± 1.63 | 0.07 | 1.39 ± 0.93 | **0.03**, 0.53 |
| **IFN-γ/IL-10** | 1.55 ± 0.65 | 1.03 ± 0.73 | **0.02** | 0.68 ± 0.45 | **0.01**, 0.36 |
| **IL-17A/IL-10** | 4.14 ± 1.95 | 1.94 ± 1.52 | **0.01** | 2.40 ± 1.64 | **0.05**, 0.55 |
| **Th17/Th2** | 3.78 ± 2.21 | 1.77 ± 1.49 | **0.03** | 2.62 ± 2.19 | 0.36, 0.41 |

| CSF cytokine ratios | GR | PR | p | noRx | p (vs GR, PR) |
|---|---|---|---|---|---|
| **Th1/Th2** | 2.37 ± 1.58 | 2.36 ± 1.43 | 0.92 | 2.55 ± 0.66 | 0.23, 0.75 |
| **Th1/Th17** | 0.81 ± 0.40 | 0.65 ± 0.49 | 0.33 | 1.36 ± 1.46 | 0.88, 0.28 |
| **Type 1[a]/Type 2** | 2.93 ± 1.51 | 2.67 ± 1.66 | 0.67 | 3.82 ± 1.56 | 0.20, 0.14 |
| **Type 1[b]/Type 2** | 2.55 ± 1.35 | 3.55 ± 3.90 | 0.91 | 3.63 ± 1.42 | 0.21, 0.36 |
| **IFN-γ/IL-10** | 1.75 ± 1.39 | 1.30 ± 1.22 | 0.27 | 1.42 ± 1.10 | 0.58, 0.86 |
| **IL-17A/IL-10** | 4.06 ± 2.67 | 2.98 ± 2.31 | 0.29 | 4.77 ± 3.03 | 0.47, 0.18 |
| **Th17/Th2** | 2.25 ± 2.00 | 4.21 ±3.69 | 0.16 | 3.59 ± 2.23 | 0.10, 0.96 |

Data are given as mean ± SD; [a]IL-2 values included; [b]IL-2 values omitted; numbers in bold denote statistical significance.
